# Alteration of Trace Elements during Pathogenesis of *N*-Nitrosodimethylamine Induced Hepatic Fibrosis

**DOI:** 10.1038/s41598-018-37516-4

**Published:** 2019-01-24

**Authors:** Joseph George, Mikihiro Tsutsumi, Mutsumi Tsuchishima

**Affiliations:** Department of Hepatology, Kanazawa Medical University, Uchinada, Ishikawa 920-0293 Japan

## Abstract

The biochemical abnormalities and oxidative stress during pathogenesis of hepatic fibrosis could lead to alteration of trace elements. We studied the alteration of major trace elements during the pathogenesis of *N*-nitrosodimethylamine (NDMA)-induced hepatic fibrosis in rats. The biochemical and pathological indices of liver functions and hepatic fibrosis were evaluated. Serum and liver levels of copper, iron and zinc were determined using atomic absorption spectrophotometry. Cobalt, manganese, and molybdenum in the serum and liver were estimated by inductively coupled plasma mass spectrometry. Serial administrations of NDMA resulted in decreased serum albumin, biochemical abnormalities, increase of total liver collagen, and well-developed fibrosis and early cirrhosis. Serum and liver zinc content significantly decreased on all the days following NDMA administration. When copper and molybdenum markedly increased in the serum, liver molybdenum decreased dramatically. Both iron and manganese content significantly increased in the liver following NDMA-induced fibrosis. The results of the present study indicate that alteration of trace elements during pathogenesis of hepatic fibrosis is due to metabolic imbalance, biochemical abnormalities, decreased serum albumin, and ascites following NDMA-induced liver injury. The modulation of trace elements during hepatic fibrosis could play a prominent role in progression of the disease.

## Introduction

Hepatic fibrosis and alcoholic cirrhosis are conditions associated with excessive synthesis and deposition of connective tissue components, especially fibrillar collagens in the parenchyma of hepatic tissue^1–4^. The pathogenesis of hepatic fibrosis is a very complex and dynamic process involving several cell types, cytokines, and growth factors and repeated wound healing that lead to hepatic scarring and hardening of liver^5–8^. Both hepatic fibrosis and alcoholic cirrhosis are debilitating and life threatening diseases and could lead to the development of hepatocellular carcinoma^9,10^. Since many trace elements are integral component of the enzymes involved in connective tissue metabolism, alteration of trace elements in the serum and liver may have a relationship with the pathogenesis of the liver diseases^11,12^.

Many trace elements are actively involved in biosynthesis and maturation of collagen and related structural proteins. Elements also play important roles as integrate part or cofactors for several enzymes involved in cellular antioxidant defense. Copper is essential for the normal cross-linking of collagen^13,14^. Lysyl oxidase, a tightly bound copper containing enzyme, involved in the oxidative deamination of ε-amino groups of certain lysine and hydroxylysine residues of collagen, yielding reactive aldehydes for cross-linking. Cobalt is an intrinsic part of vitamin B_12_ and required for the function of several enzymes^15^. Iron is important in connective tissue metabolism and is necessary for the activity of prolyl hydroxylase and lysyl hydroxylase, enzymes involved in the hydroxylation of proline and lysine residues respectively, during biosynthesis of collagen^16,17^. Manganese is an essential cofactor for the activity of hydroxylysyl galactosyltransferase and galactosylhydroxylysyl glucosyltransferase, enzymes responsible for the attachment of galactose and glucose residues to hydroxylysine^18,19^. Manganese is required for the normal function of many enzymes, including pyruvate carboxylase, and superoxide dismutase. It is an important trace element involved in the biosynthesis of oligosaccharides, glycoproteins, and proteoglycans^20^. Molybdenum is required for the function of xanthine, aldehyde, and sulfite oxidases^21^. Several matrix metalloproteinases, the primary collagen degrading enzymes, constitute zinc as an integral part of the enzyme protein^22^.

It was observed that HCV-related cirrhotic patients have significant decrease of blood levels of zinc and selenium, independent of nutritional status^23^. The increased incidence of aphthous stomatitis in patients with HCV infection could be due to decreased serum zinc levels^24^. Abnormal zinc metabolism has been well documented in alcoholic liver cirrhosis^25,26^. A significantly increased serum copper and manganese levels were noticed in patients with chronic liver diseases^27^. However, the pathophysiological correlation between modulation of trace elements and pathogenesis of hepatic fibrosis is not clear. Furthermore, very little information is available concerning changes in the liver levels of trace elements in hepatic fibrosis. In the current investigation, we measured serum and liver levels of trace elements, cobalt, copper, iron, manganese, molybdenum, and zinc during the pathogenesis of *N*-nitrosodimethylamine (NDMA)-induced hepatic fibrosis in rats employing atomic absorption spectrophotometry and inductively coupled plasma-mass spectrometry.

## Materials and Methods

### Animal model for hepatic fibrosis

Adult male albino rats of Wistar strain at the age of around 3 months and weighing between 180–200 g were used for induction of hepatic fibrosis. The animals were maintained in air-conditioned animal house under 12-h light/12-h dark cycles with commercial rat feed pellets and water available ad libitum. All the animal experimental methods were carried out in accordance with relevant guidelines and regulations and followed the *Guide for the Care and Use of Laboratory Animals* prepared by the National Academy of Sciences and published by the US National Institutes of Health (NIH Publication No. 86-23, revised 1996). All the animal experimental protocols were approved by the ethical committee at Kanazawa Medical University for the care and use of laboratory animals.

### Induction of hepatic fibrosis in rats

Hepatic fibrosis was induced by serial administrations of *N*-nitrosodimethylamine (NDMA) (Sigma-Aldrich, St. Louis, MO, USA) in a dose of 10 mg (10 μL diluted to 1 mL with 0.15 mol/L sterile NaCl)/kg body weight on three consecutive days of a week over a period of three weeks^28^. The dosage of NDMA, course of the treatment, and duration of the experiment have been standardized previously in such a way to develop well-formed fibrosis and early cirrhosis to study the events associated with the pathogenesis of hepatic fibrosis^29–31^. Control animals received sterile NaCl without NDMA. Body weight and behavioral changes of the experimental animals were monitored throughout the study. Treated and control animals were sacrificed on days 7, 14, and 21 from the beginning of exposure. The animals were anaesthetized with diethyl ether and blood was collected from right jugular vein. Serum was separated in the conventional way and stored in screw capped polypropylene serum vials at −80 °C until assayed. The livers were rapidly removed, washed in cold phosphate buffered saline, and weighed in the wet state after blotting off water. A median lobe of 3 mm thick was cut and instantly fixed in 10% phosphate-buffered formalin for histopathological studies. Another portion of the liver was frozen at −80 °C for biochemical analyses. Extreme care was taken to avoid any metal contamination of either liver tissue or serum samples at every point of handling.

### Measurement of liver function parameters

All major liver function indices such as alanine transaminase (ALT), aspartate transaminase (AST), γ-glutamyl transpeptidase (γ-GT), alkaline phosphatase (ALP), total bilirubin, albumin, and globulins were determined in the serum samples by conventional spectrophotometric methods.

### Determination of protein, hydroxyproline, and collagen content in the liver

The total protein present in the liver tissue was quantified by the method of Lowry *et al*.^32^. Hydroxyproline content in the liver tissue was determined as described before^33^ using *L-*hydroxyproline (Sigma-Aldrich, St. Louis, MO, USA) as standard. The total collagen content in the liver tissue was calculated by multiplying the hydroxyproline content by the factor 7.46 as reported previously^34^.

### Histopathological assessment of NDMA-induced hepatic fibrosis

The degree of NDMA-induced hepatic fibrosis was evaluated histopathologically after Hematoxylin & Eosin (H&E) and Masson’s trichrome staining of paraffin liver sections employing conventional methods. The stained sections were examined using an Olympus BX51 microscope (Olympus Corp, Tokyo, Japan) attached with a digital camera and photographed.

### Immunohistochemical staining for α-smooth muscle actin

The immunohistochemical staining for α-smooth muscle actin (α-SMA) was carried out on paraffin-embedded liver sections using a universal staining kit (#85-9943; Invitrogen, Carlsbad, CA, USA). The deparaffinized and hydrated liver sections were treated with a primary antibody against α-SMA (#412021, Nichirei Biosciences, Tokyo, Japan). The slides were washed 3–5 times in cold phosphate-buffered saline (PBS) and incubated with biotinylated secondary antibody for 1 h at room temperature. It was washed again, treated with streptavidin-conjugated horseradish peroxidase, and incubated for another 1 h. The color was developed after treatment with 3% 3-amino-9-ethylcarbazole (AEC) for 10 min. The stained sections were counterstained with Mayer’s haematoxylin and examined under a microscope (Olympus BX51, Tokyo, Japan) attached with a digital camera (Olympus DP71) and photographed.

### Quantitative real-time PCR for α-smooth muscle actin and collagen type I

Quantitative real-time PCR (qPCR) was carried out for α-smooth muscle actin and collagen type I mRNA to evaluate the changes in gene expression during the pathogenesis of NDMA-induced hepatic fibrosis. The liver tissue was flash frozen in liquid nitrogen and stored at −80 °C. Total RNA was isolated employing PureLink RNA Mini Kit (#12183018 A, Thermo Fisher) in combination with Trizol reagent as per manufacturer’s instructions. The gene specific primers for α-SMA, type I collagen (α1 chain), and GAPDH were designed using Beacon Designer software (Premier Biosoft International, Palo Alto, CA). The selected forward primer sequence for rat α-SMA (NM_031004.2) began at nucleotide 364 (5′-CATCCGACCTTGCTAACGGA-3′) and the reverse at 729 (3′-GTCCAGAGCGACATAGCACA-5′). The forward primer sequence for collagen type I (α1 chain) (NM_053304.1) began at nucleotide 3140 (5′-AAGGCTCCCCTGGAAGAGAT-3′) and the reverse sequence at 4114 (3′-CAGGATCGGAACCTTCGCTT-5′). The forward primer sequence for the housekeeping gene GAPDH (NM_017008.4) began at nucleotide 306 (5′-GCGAGATCCCGCTAACATCA-3′) and for the reverse primer at 1153 (3′-GATGGGGACTCCTCAGCAAC-5′). The cDNA was synthesized using 1–2 μg of total RNA with Sprint RT 8-well strips (#639532, Clontech, Mountain View, CA) in a total volume of 20 μl in RNAse-free water at 42 °C for 60 min. The synthesized cDNA was diluted to 20 fold with RNAse-DNAse-free water. The qPCR reaction was performed using 5 µl of diluted cDNA and 5 µl of FastStart SYBR Green master mix (Roche Applied Science, Indianapolis, IN) with gene specific primers in a 384 well plate (total volume 10 µl) on ABI 7500 Real-Time PCR system (Applied Biosystems, Foster City, CA). All the samples were run in triplicates. The qPCR parameters were set as follows; denature 1 cycle, amplification 45 cycles, melting curve 1 cycle and cooling 1 cycle. All the data were normalized to GAPDH gene.

### Extraction of elements from the liver for analysis by Atomic Absorption Spectrophotometry and Inductively Coupled Plasma Mass Spectrometry

Since Atomic Absorption Spectrophotometry (AAS) and Inductively Coupled Plasma-Mass Spectrometry (ICPMS) are very sensitive methods for determination of elements, all possible measures were taken to avoid contamination from elements at every processing and handling step. Exactly 100 mg frozen liver tissue was weighed and dried in redistilled acetone. It was predigested with 2 ml of redistilled concentrated nitric acid in a very clean beaker with a cover glass at 110–120 °C until it turned pale yellow. After cooling, 2 ml of quartz-distilled 70% perchloric acid was added and digested at about 180 °C until it became clear and colorless solution. It was cooled and made up to 10 ml with penta distilled deionized (Milli-Q18.2 MΩ) water (PDW) to obtain a concentration of 10 mg original liver tissue/ml. This solution was further diluted to obtain a suitable analytical range for each element present in the liver tissue and used for the analysis by AAS and ICPMS. Two blanks were similarly treated and incorporated in the assay. All AAS measurements were carried out on a Varian Techtron model AA-1475 double beam atomic absorption spectrophotometer (Varian Instrument Division, Palo Alto, California) using air-acetylene flame. All ICPMS measurements were carried out on a Perkin-Elmer SCIEX^R^ ELAN^TM^ 250 ICP-MS (Amherst, New York).

### Standard and sample preparations for AAS and ICPMS

All required standard solutions for AAS and ICPMS were prepared and diluted in Corning polypropylene centrifuge tubes (Corning, New York). All the tubes were rinsed three times with PDW and after the third rinsing; a prerun was carried out for each element after appropriate instrumental settings. Tubes that showed reading for any element were discarded. The PDW used for the study was also tested for every element studied and made sure that it did not produce reading for any element. All the serum and liver samples were diluted with pretested PDW in screw capped polypropylene tubes.

### Determination of Copper, Zinc, and Iron by AAS

Copper, iron, and zinc present in serum and liver samples were quantified using AAS. The parameters used for estimation of copper, iron, and zinc by AAS are depicted in Table 1. In AAS, hollow cathode lamps are used to produce monochromatic light specific to each element that produce extremely sharp resonance lines of 0.001–0.01A° width. The resonance lines are characteristic and specific for each element and no two elements possess an identical resonance line. When a sample is introduced into the flame, the elements are vaporized as free atoms and the monochromatic light from the hollow cathode lamp of a particular element is passed, it is absorbed by the atoms of that element whose resonance line is identical with the light source. The amount of light absorbed is quantified by a detector against a known standard.

**Table 1:** Parameters used for estimation of copper, iron, and zinc in serum and liver samples by atomic absorption spectrophotometry.

Trace element	Cu	Fe	Zn
Wavelength (nm)	324.7	248.3	213.9
Lamp current (mA)	3.5	5	5
Slit width (nm)	0.5	0.2	1
Fuel	Acetylene	Acetylene	Acetylene
Oxidant	Air	Air	Air
Standard range (µg/ml)	1–3	1–3	0.2–0.6

### Estimation of copper in serum and liver

The copper content in serum and liver was determined by the method described by George *et al*.^35^. For determination of copper, the serum samples were diluted 1:1 with PDW. Even though, copper concentration in serum is within the suitable analytical range of AAS, a minimum of double dilution of serum was necessary to prevent protein obstruction of the burner head and atomizer. Since the copper level in the liver tissue is very low, the liver extracts (10 mg/ml) were aspirated directly without any dilution. Air-acetylene flame was used for the estimation of copper. A copper hollow cathode lamp with lamp current of 3.5 mA was used to produce copper resonance line. The monochromator was set at 324.7 nm with a spectral band pass to 0.5 nm. Copper standard solution was prepared by dissolving 1 g of 99.99% pure copper metal strip in a minimum volume of 1:1 ultrapure nitric acid and diluted to 1 liter with PDW to give 1000 µg/ml copper. The working standards were prepared at concentrations of 1, 2, and 3 μg/ml of copper.

### Estimation of zinc in serum and liver

Zinc present in serum and liver samples was determined by the method of Kiilerich *et al*.^36^. The serum samples were diluted to 1:4 and the liver extracts (10 mg/ml) were diluted 10-fold with zinc free PDW in polypropylene tubes. A zinc hollow cathode lamp (Varian Techtron) was used for the measurement with a lamp current of 5 mA. The wavelength was set at 213.9 nm with a spectral band pass of 1.0 nm. For standard, 1 g of specpure (99.99%) zinc metal granules were dissolved in 40 ml of 1:1 zinc free ultrapure hydrochloric acid and diluted to 1 liter with PDW to provide 1000 μg/ml zinc. Working standards were prepared at concentrations of 0.2, 0.4, and 0.6 μg/ml of zinc with zinc free PDW.

### Estimation of iron in serum and liver

The method for estimation of iron present in serum and liver samples was standardized in our laboratory. The serum samples were diluted 1:2 and the liver extracts (10 mg/ml) were diluted 5 fold with PDW in polypropylene tubes. The required iron resonance line was produced using an iron hollow cathode lamp (Varian Techtron) with a lamp current of 5 mA. The wavelength was adjusted to 248.3 nm and the spectral band pass (slit width) to 0.2 nm. Iron standard solution was prepared by dissolving 1.0 g of specpure (99.9%) iron metal strip in 20 ml of 1:1 redistilled hydrochloric acid and diluted to 1 liter with PDW to provide 1000 μg/ml iron. The optimum range of working standards were prepared at concentrations of 1, 2, and 3 μg/ml of iron with PDW.

### Determination of cobalt, manganese, and molybdenum by ICPMS

The trace elements cobalt, manganese, and molybdenum present in serum and liver samples were determined using ICPMS by the method of Vanhoe *et al*.^37^. In ICPMS, inductively coupled plasma (ICP) acts as the ion source for the mass spectrometer. ICP is electrodeless argon plasma formed at atmospheric pressure by means of induction heating caused by a radio frequency electromagnetic field. Generally, the sample solutions are sprayed into the ICP with a nebulizer. Because of the high temperature of the plasma, dissolved solids are vaporized, atomized, and ionized. Ions are extracted into the mass spectrometer by means of differential pumping through a sampler and skimmer. Mass analysis is done by a quadrupole mass analyzer and the ions are detected by channel electron multiplier. Based on the counts of elements in known standards, the amount of a particular element present in the unknown samples can be determined.

Cobalt, manganese, and molybdenum present in the serum and liver samples were simultaneously determined after appropriate instrumental settings on the computer. Both serum (200 μl) and liver samples (10 mg/ml) were diluted to 10-fold with PDW in polypropylene tubes. An internal standard cesium was added to a final concentration of 10 ng/ml to all samples in order to reduce the instrumental drift during operation. The argon flow was adjusted to 12 L/min for coolant and 1.4 L/min as auxiliary. The nebulizer argon flow was 0.4 liter/min with a nebulizer pressure of 38 psi. The sampling depth was 23 mm. The readings were recorded in sequential mode with a measurement time interval of 0.5 seconds and with three measurements per peak. All sample measurements were repeated 10 times and the mean value was taken.

Cobalt standard was prepared by dissolving 1 g of specpure cobalt metal strip in a minimum volume 1:1 redistilled nitric acid, diluted to 1 liter with PDW, and the acidity was adjusted to 1 N. The manganese standard was prepared by dissolving 1 g of specpure manganese metal strip in 1:1 redistilled nitric acid and diluted to 1 liter to provide 1000 μg/ml manganese. For molybdenum standard, 1 g of specpure molybdenum was dissolved in 1:3 mixture of ultrapure concentrated nitric and hydrochloric acid, diluted to 1 liter to give 1000 μg/ml molybdenum and the acidity was maintained at 1 N. The calibration curve was constructed in the range of 0.4–4 ng/ml in the case of cobalt and molybdenum and 4–40 ng/ml for manganese. All standards were prepared with 10 ng/ml cesium as internal standard. A higher concentration range of standard curve was prepared for manganese due to the presence of higher amount of the metal in serum and liver.

### Statistical analysis

The mean and standard deviation were calculated for all the data. The results were statistically evaluated by using one-way analysis of variance (ANOVA). The control mean values were compared with the experimental mean values on days 7, 14, and 21 using the least significant difference method. A value of *p* < 0.05 was considered as statistically significant. Pearson’s correlation coefficient was used to study the linear relationship between the alteration of serum and liver levels of certain elements.

## Results

### Alteration of biochemical and liver function parameters during pathogenesis of NDMA-induced hepatic fibrosis

Alteration of various biochemical and liver function parameters monitored during the pathogenesis of NDMA-induced hepatic fibrosis is presented in Table 2. A significant decrease (*p* < 0.001) was observed in animal body weight and liver weight on days 14 and 21 after the start of NDMA administration. The total collagen present in the liver tissue, measured in terms of hydroxyproline content, increased significantly (*p* < 0.001) on all the days evaluated following NDMA administration (Table 2). All the major liver function indices such as ALT, AST, γ-GT, ALP, and total bilirubin in the serum were markedly increased (*p* < 0.001) on days 14 and 21 after the start of NDMA administration (Table 2). In the case of γ-GT, there was significant elevation on day 7 also. The total protein content in the liver tissue was decreased significantly on all the days evaluated. A marked reduction (*p* < 0.001) was noticed in serum albumin levels on 7th, 14th, and 21st day from the onset of NDMA treatment (Table 2). Hypoalbuminaemia is a strong indicator of decreased liver function and ascites. Serum globulins were significantly increased on all the days following NDMA administration (Table 2).

**Table 2:** Biochemical and liver function parameters during the pathogenesis of NDMA-induced hepatic fibrosis in rats.

Parameters assayed	Control (*n* = 12)	Day 7 (*n* = 12)	Day 14 (*n* = 12)	Day 21 (*n* = 12)
Body weight (g)	195.26 ± 7.32	187.45 ± 8.21	168.42 ± 10.42**	146.32 ± 9.32**
Liver weight (g)	7.72 ± 0.56	7.39 ± 0.63	4.78 ± 0.43**	3.46 ± 0.36**
Total liver collagen^a^	1.02 ± 0.07	1.94 ± 0.13**	2.98 ± 0.17**	4.03 ± 0.36**
Serum ALT (units/mL)	45.23 ± 3.34	85.86 ± 5.07	145.62 ± 9.34**	245.35 ± 17.24**
Serum AST (units/mL)	93.74 ± 5.03	158.45 ± 8.46	255.31 ± 23.32**	455.45 ± 38.46**
Serum y-GT^b^	15.21 ± 0.69	33.31 ± 1.95**	43.35 ± 4.21**	38.29 ± 1.62**
Serum ALP^c^	645.11 ± 21.32	635.2 ± 38.20*	845.34 ± 45.34**	943.93 ± 57.77**
Total bilirubin (mg/100 mL)	0.55 ± 0.03	0.78 ± 0.05	1.24 ± 0.11**	1.83 ± 0.32**
Total liver protein^a^	151.62 ± 8.78	137.28 ± 10.46*	129.58 ± 12.62**	123.17 ± 10.23**
Serum albumin (g/100 mL)	3.98 ± 0.12	3.65 ± 0.11**	2.76 ± 0.13**	2.46 ± 0.09**
Serum globulin (g/100 mL)	3.19 ± 0.13	3.89 ± 0.21*	4.79 ± 0.22**	4.68 ± 0.19**

Values are mean ± standard deviation.

^a^Expressed as milligrams per gram of liver wet weight.

^b^Values are expressed as nanomoles of *p*-nitroaniline liberated/min/mL serum.

^c^Values are expressed as nanomoles of phenol liberated/min/mL serum.

*p < 0.01 and **p < 0.001 (by ANOVA).

### Assessment of the degree of NDMA-induced hepatic fibrosis in rat liver

The course of the pathogenesis of NDMA-induced hepatic fibrosis in rat liver was assessed histopathologically employing Hematoxylin & Eosin (H&E) and Masson’s trichrome staining. Immunohistochemical staining for α-smooth muscle actin (α-SMA) was carried out to demonstrate activated stellate cells that contribute excessive synthesis and deposition of fibrillar collagens in the liver.

**Figure 1:**
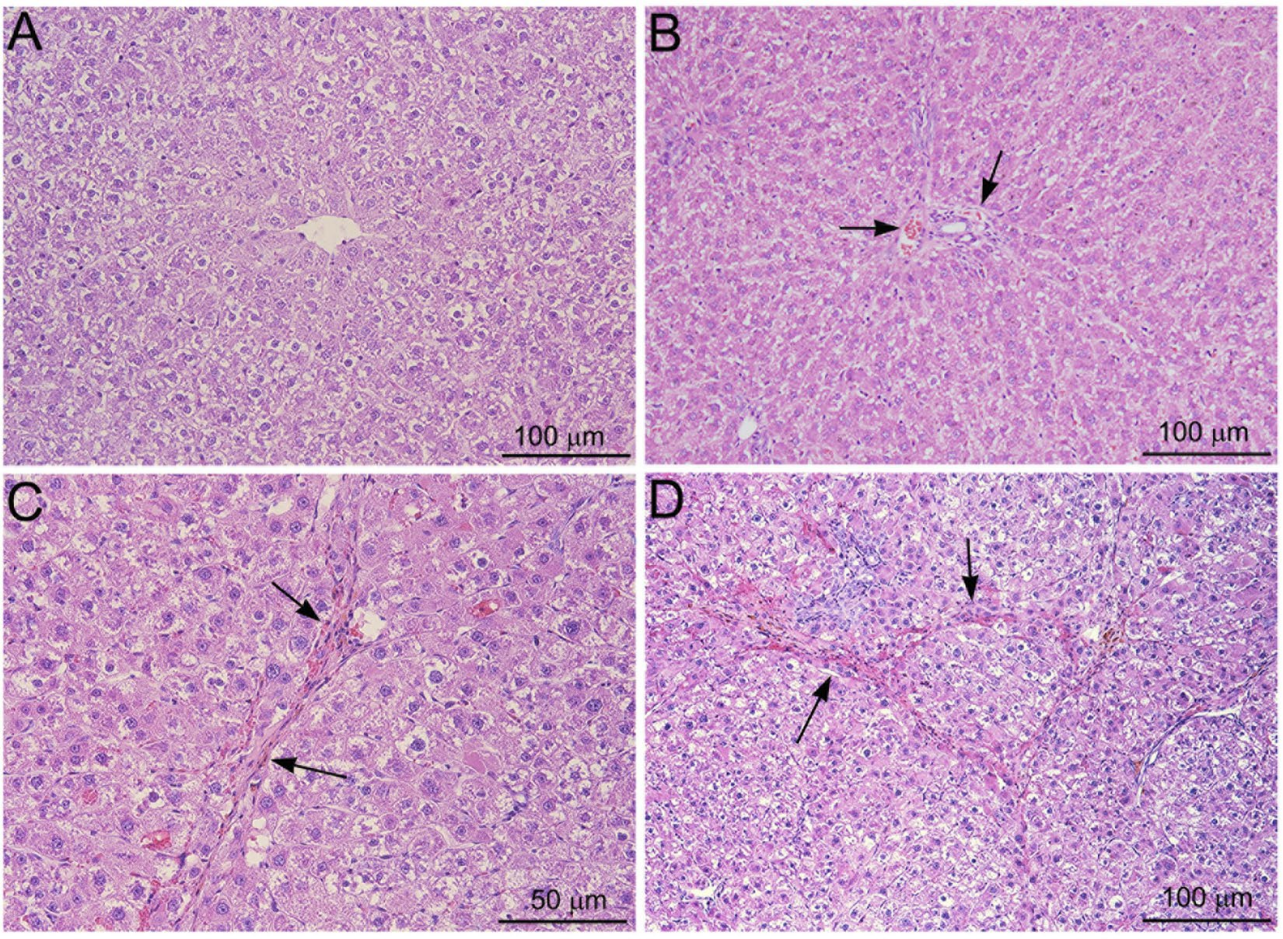
Hematoxylin and Eosin staining of rat liver sections following serial administrations of NDMA. (**A**) Normal liver. (**B**) NDMA Day 7. Dilation of sinusoids and focal hemorrhage (arrows). (**C**) NDMA Day 14. Development of fibrosis and formation of thick collagen fibers (arrows). (**D**) NDMA Day 21. Well developed fibrosis and early cirrhosis with deposition of thick collagen fibers (arrows). Original magnification, ×100 for (**A**,**B** and **D**); ×200 for (**C**).

Images of H&E staining in rat liver sections following NDMA administrations are presented in Fig. 1. The untreated rat livers depicted normal lobular architecture with central veins and radiating hepatic cords (Fig. 1A). On day 7 following NDMA treatment, centrilobular necrosis and focal hemorrhage were present (arrows). There was significant dilatation of central veins and sinusoids (Fig. 1B). On day 14, marked fibrosis with deposition of collagen fibers was conspicuous (arrows) (Fig. 1C). On day 21, there was well-developed fibrosis and early cirrhosis with deposition of extensive thick collagen fibers in the hepatic parenchyma (arrows) (Fig. 1D).

### Masson’s trichrome staining for mature collagen fibers

**Figure 2:**
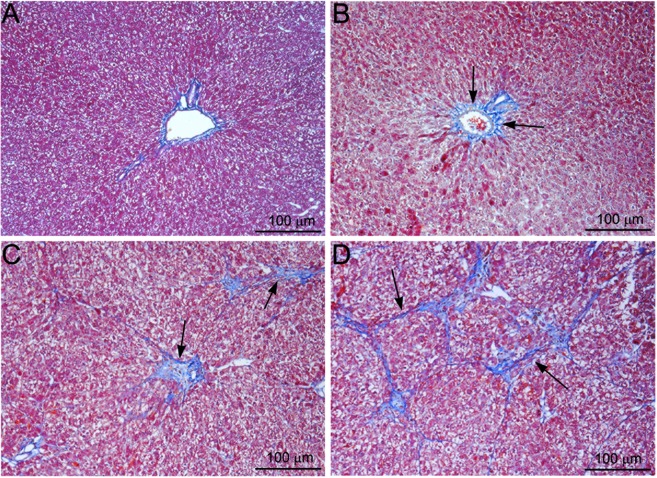
Masson’s trichrome staining for collagen in rat liver sections following serial administrations of NDMA. The animals are injected with NDMA in a dose of 10 mg/kg body weight on three consecutive days of a week for three weeks. (**A**) Normal liver (**B**) NDMA Day 7. Pericentral fibrosis and early deposition of fine collagen fibers (arrows). (**C**) NDMA Day 14. Formation of bridging fibrosis between central vein and portal triads and deposition of thick collagen fibers (arrows). (**D**) NDMA Day 21. Well developed hepatic fibrosis and early cirrhosis. Deposition of extensive thick collagen fibers in the hepatic parenchyma (arrows). Original magnification, ×100.

Masson’s trichrome staining for mature collagen is depicted in Fig. 2. Trichrome staining for collagen was absent in normal livers except in central veins and portal triads (Fig. 2A). On day 7 following NDMA treatment, fibrosis was initiated with the deposition of collagen fibers in pericentral and periportal areas (arrows) (Fig. 2B). On day 14, there was intense deposition of thick collagen fibers with early bridging between central vein and portal triads (arrows) (Fig. 2C). On day 21, there was well developed fibrosis and early cirrhosis with extensive deposition of thick collagen fibers between central vein and portal triads. The bridging fibrosis initiated the formation of multiple porto-central bridging septa leading to cirrhosis (arrows) (Fig. 2D).

### Immunohistochemical staining for activated stellate cells

**Figure 3:**
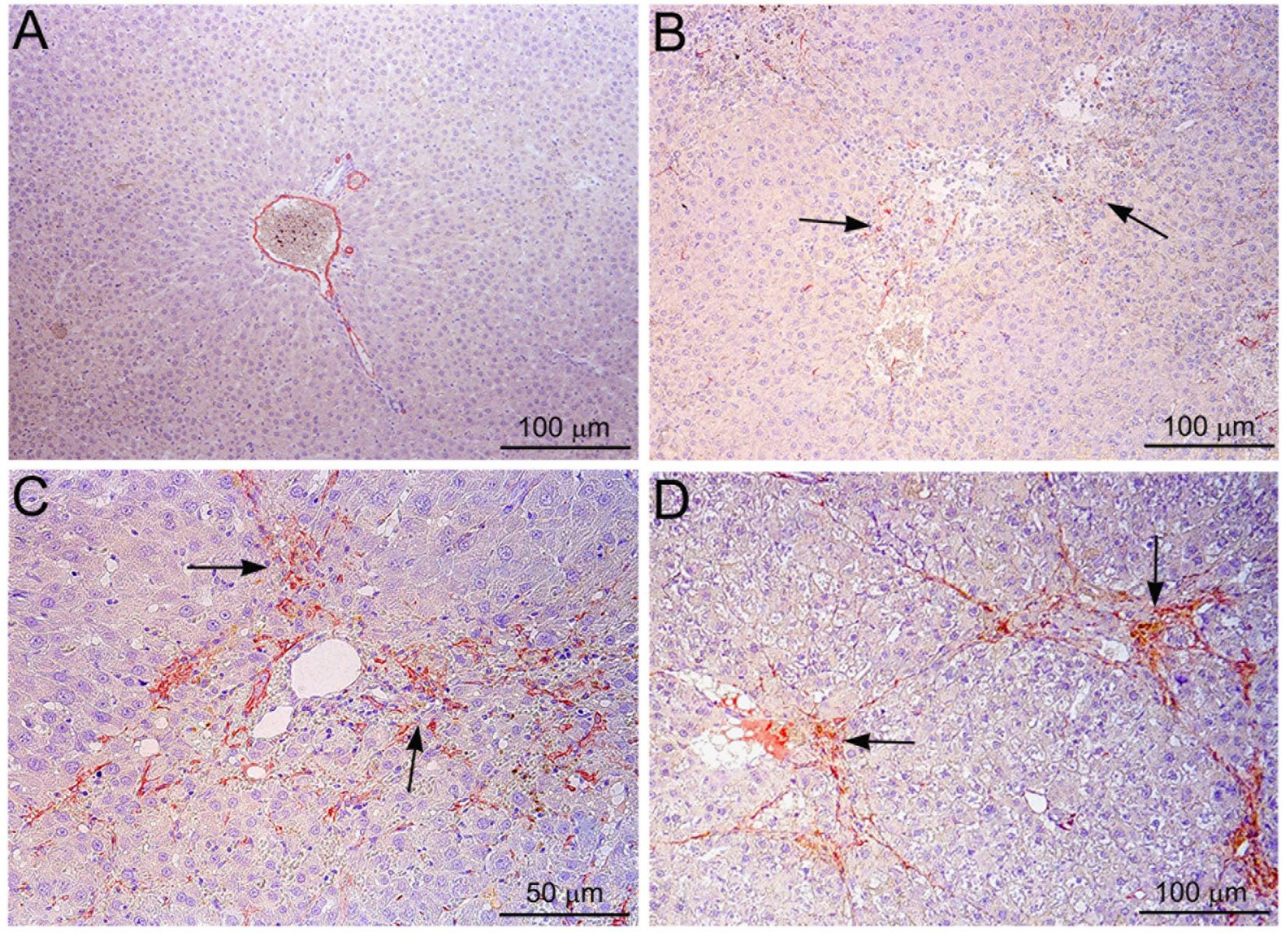
Immunohistochemical staining of α−smooth muscle actin (α−SMA) in rat liver sections demonstrating activated hepatic stellate cells following serial administrations of NDMA. (**A**) Normal liver. Absence of α−SMA staining in the hepatic parenchyma. (**B**) NDMA 7 days. Positive staining for α−SMA in necrotic areas indicating activated stellate cells (arrows). (**C**) NDMA 14 days. Abundant staining of α−SMA indicating enormous number of activated stellate cells, especially in necrotic zone (arrows). (**D**) NDMA 21 days. Marked staining of α−SMA demonstrating activated stellate cells in fibrotic areas (arrows). Original magnification, ×100 for (**A**,**B** and **D**); ×200 for (**C**).

Immunohistochemical staining for α-SMA was carried out to demonstrate the activation of hepatic stellate cells after treatment with NDMA and the data are presented in Fig. 3. The staining for α-SMA was completely absent in the hepatic parenchyma in control livers (Fig. 3A). On day 7 following NDMA treatment, positive staining for α-SMA was present in necrotic areas indicating activation of hepatic stellate cells (arrows) (Fig. 3B). On day 14, there was remarkable staining of α-SMA indicating enormous number of activated stellate cells, especially in the necrotic zone (arrows) (Fig. 3C). On day 21, the staining for α-SMA was focalized to the well-developed fibrosis areas (arrows). The staining was very strong and conspicuous indicating the association of activated stellate and pathogenesis of hepatic fibrosis (Fig. 3D). This indicates that the activated hepatic stellate cells synthesize and deposit collagens in the liver during fibrogenesis.

### Changes in gene expression of α-smooth muscle actin and collagen type I

**Figure 4:**
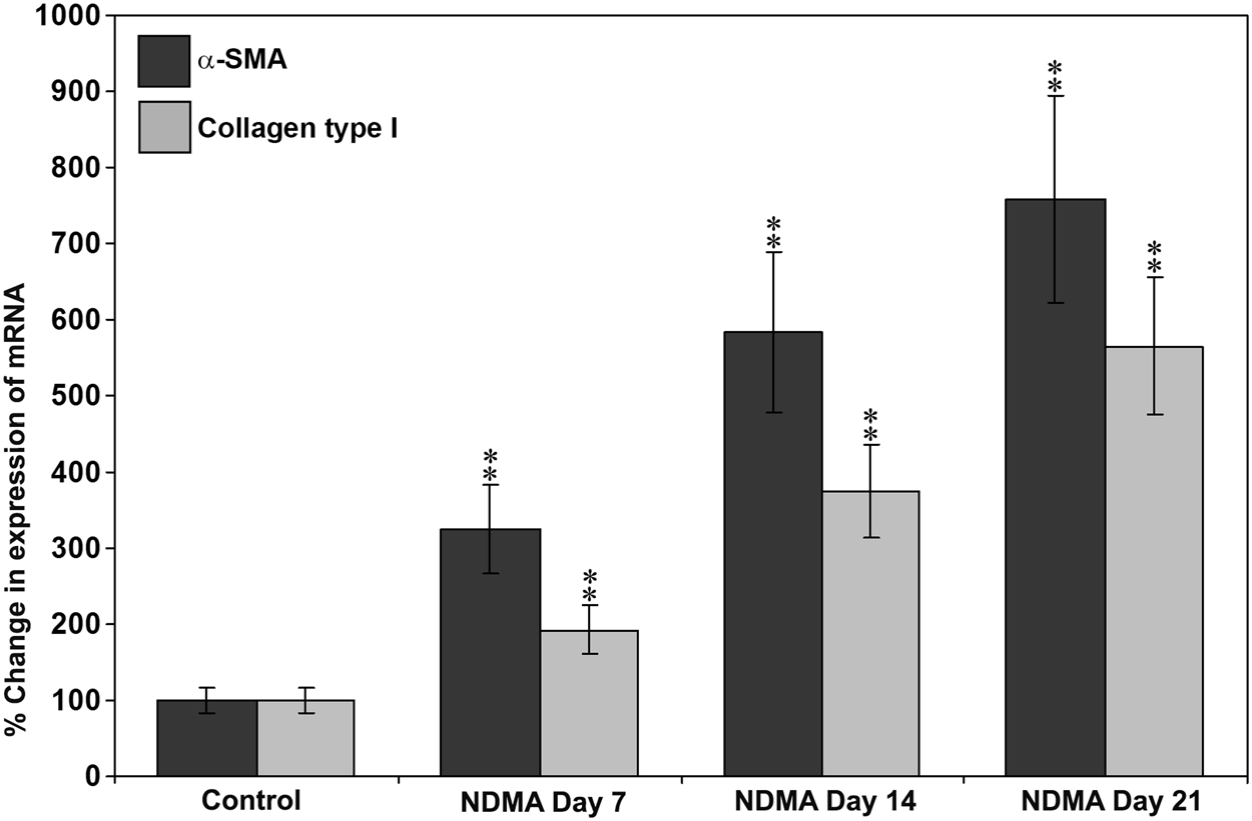
Expression of α−SMA and collagen type I (α1 chain) mRNA during the pathogenesis of NDMA-induced hepatic fibrosis in rats. All the data were normalized to GAPDH gene. The data are mean ± S.D. (N = 6). **p < 0.001 (by ANOVA); NDMA-treated versus untreated control rats.

The results of qPCR performed to examine the changes in gene expression of α-SMA and collagen type I (α1 chain) during the pathogenesis of NDMA-induced hepatic fibrosis are presented in Fig. 4. A steady state increase in the mRNA levels of α-SMA was observed from day 7 through day 21 during NDMA treatment with a maximum of 7.58 fold increase on day 21. Similarly, collagen type I (α1 chain) mRNA elevated markedly and significantly on days 7, 14, and 21 with a maximum increase of 5.44 fold on day 21 compared to untreated control rats.

### Serum and liver concentrations of cobalt

Cobalt levels in serum and liver during the pathogenesis of NDMA-induced hepatic fibrosis are presented in Tables 3 and 4, respectively. Cobalt, an integral component of vitamin B_12_, depicted a reduction (*p* < 0.01) in serum on day 21 of NDMA treatment. Cobalt levels in the serum were not significantly different on other days studied. The liver cobalt levels did not show any significant alteration on all the days evaluated indicating a normal liver vitamin B_12_ levels during the pathogenesis of hepatic fibrosis.

**Table 3:** Trace elements in serum during pathogenesis of NDMA-induced hepatic fibrosis in rats.

Trace element	Control (*n* = 12)	Day 7 (*n* = 12)	Day 14 (*n* = 12)	Day 21 (*n* = 12)
Cobalt (ng/ml)	3.28 ± 0.28	3.44 ± 0.24	2.90 ± 0.24	2.20 ± 0.21*
Copper (µg/100 ml)	98.46 ± 3.65	112.32 ± 4.61*	123.90 ± 5.78**	135.72 ± 6.71**
Iron (µg/100 ml)	237.16 ± 10.39	232.50 ± 16.75	224.20 ± 12.92	227.65 ± 14.80
Manganese (ng/ml)	42.50 ± 3.96	68.85 ± 5.72*	63.53 ± 4.34*	65.62 ± 4.62*
Molybdenum (ng/ml)	35.79 ± 2.88	38.54 ± 3.01	61.09 ± 5.65**	67.15 ± 5.80**
Zinc (µg/100 ml)	122.27 ± 5.75	87.10 ± 3.44**	75.24 ± 3.73**	58.50 ± 3.54**

Values are mean ± standard deviation.

*p < 0.01 and **p < 0.001 (by ANOVA).

**Table 4:** Trace elements in the liver during pathogenesis of NDMA-induced hepatic fibrosis in rats.

Trace element	Control (*n* = 12)	Day 7 (*n* = 12)	Day 14 (*n* = 12)	Day 21 (*n* = 12)
Cobalt (µg/g)	0.54 ± 0.06	0.61 ± 0.07	0.47 ± 0.05	0.52 ± 0.04
Copper (µg/g)	4.52 ± 0.25	4.98 ± 0.30	5.82 ± 0.38*	6.45 ± 0.46**
Iron (µg/g)	113.60 ± 6.25	182.03 ± 12.91*	261.33 ± 17.18**	334.93 ± 20.90**
Manganese (µg/g)	3.21 ± 0.22	5.24 ± 0.41*	6.04 ± 0.54*	8.22 ± 0.72**
Molybdenum (µg/g)	2.18 ± 0.18	2.23 ± 0.21	0.72 ± 0.09**	0.56 ± 0.06**
Zinc (µg/g)	55.25 ± 3.92	41.12 ± 2.95**	32.16 ± 2.64**	27.37 ± 2.36**

Values are mean ± standard deviation.

All values are expressed as µg/g liver wet weight.

*p < 0.01 and **p < 0.001 (by ANOVA).

### Serum and liver concentrations of copper

Copper levels in serum and liver are presented in Tables 3 and 4, respectively. There was significant increase in serum copper levels on 7th, 14th, and 21st days after the start of NDMA administration (Table 3). The increase was gradual and the maximum elevation was noticed on day 21. Liver copper levels were also increased significantly on 14th and 21st days following NDMA treatment (Table 4).

### Serum and liver concentrations of iron

Iron concentrations in serum and liver during the progression of NDMA-induced hepatic fibrosis are depicted in Tables 3 and 4, respectively. Serum iron levels did not produce any significant alterations during the course of NDMA administration. However, liver iron levels were remarkably increased on 7th, 14th, and 21st days following NDMA treatment (Table 4). The increase was gradual and the maximum increase was observed on day 21, which was around 3-fold higher compared to the liver iron concentration in untreated control livers.

### Serum and liver concentrations of manganese

Tables 3 and 4 demonstrate manganese levels in serum and liver, respectively during the pathogenesis of NDMA-induced hepatic fibrosis. Serum manganese levels exhibited a significant increase (*p* < 0.01) on 7th, 14th, and 21st days after the beginning of NDMA administration (Table 3). The liver manganese levels also depicted a significant elevation on 14th and 21st days after the onset of hepatic fibrosis (Table 4). Even though, there was moderate increase in liver manganese levels on day 7 following NDMA treatment, the increase lacked significance due to high variance.

### Serum and liver concentrations of molybdenum

Molybdenum concentrations in serum and liver during the pathogenesis of NDMA-induced hepatic fibrosis are presented in Tables 3 and 4, respectively. A significant increase (*p* < 0.001) was observed in serum molybdenum levels on 14th and 21st days following NDMA administration (Table 3). In contrast, a significant and marked decrease (*p* < 0.001) was noticed in liver molybdenum concentration on days 14th and 21st during NDMA treatment (Table 4). The maximum decrease was on day 21 which was around 4-fold. A highly negative correlation (r = −0.992) was noticed between the increased serum and decreased liver molybdenum concentrations.

### Serum and liver concentrations of zinc

Zinc levels in serum and liver during the progression of NDMA-induced hepatic fibrosis are demonstrated in Tables 3 and 4, respectively. Serum zinc concentrations were significantly decreased (*p* < 0.001) on all the days studied after the start of NDMA administration (Table 3). The decrease was gradual with a maximum reduction on day 21, which was around 2-fold compared to the untreated control value. Zinc present in the liver tissue was also depleted significantly (*p* < 0.001) on 7th, 14th, and 21st days following NDMA treatment with a maximum decrease on day 21 (Table 4). A positive correlation (r = 0.992) was noticed between decreased serum and depleted liver zinc levels. A negative correlation (r = −0.98) was also observed between elevated copper and decreased zinc levels in the serum.

## Discussion

The results of the present study clearly indicated that trace elements present in serum and liver modulate remarkably during pathogenesis of NDMA-induced hepatic fibrosis. Since NDMA is exclusively metabolized by cytochrome P-450 family of enzymes, more specifically CYP2E1 present in the liver^38^, the pathogenesis of hepatic fibrosis during serial administrations of NDMA is related to the biochemical events that occur during the metabolic detoxification of NDMA. Since NDMA does not affect other organs or tissues significantly, the alteration of trace elements in the serum could be mostly related to its effect on the liver. Several other factors could interfere with the trace element concentrations in fibrotic rats compared to control rats. The other major factors are nutritional status, gastrointestinal disturbances, malabsorption, and metabolic disorders. In the present investigation, it was noticed that the food and water intake of the treated animals was comparatively less than that of the control animals. Furthermore, the animal body weight and liver weight were significantly reduced on day 14 and 21 after the start of NDMA administration. Previously we have reported abnormalities of mineral metabolism in the same model of NDMA-induced hepatic fibrosis^39^.

Data are not available regarding cobalt levels in hepatic fibrosis or liver cirrhosis either in human or in experimental animals. In the present study, serum cobalt levels decreased only on day 21 after the start of NDMA treatment and there was no alteration in the liver. Since cobalt is well absorbed in the intestine, the serum depletion could be related to the inadequate dietary intake by the fibrotic animals. Cobalt is an integral component of vitamin B_12_ and a deficiency of vitamin B_12_ was reported in alcoholic patients^40^.

Copper, an important trace element in connective tissue metabolism, was reported to be increased in the serum in patients with liver cirrhosis or alcoholic liver disease (ALD)^11,27,41^. In the present investigation also, serum copper levels increased significantly on all the days evaluated after the start of NDMA administration. It was interesting to note that in the current study, the serum copper values of control and on day 21 following NDMA treatment were very similar to the reported values of serum copper levels in controls as well as in patients with liver cirrhosis or ALD^11,27,41^. The increase of serum copper levels in experimental hepatic fibrosis and in patients with liver cirrhosis or ALD could be due to the elevation of ceruloplasmin in the serum. Ceruloplasmin contains 6 atoms of copper per molecule and constitutes more than 95% of the total serum copper^42^. Ceruloplasmin is an acute phase reactant and could be increased in all inflammatory conditions. An increased serum ceruloplasmin was noticed in patients with primary biliary cirrhosis^43^. In the present study also, total serum globulins, which constitute ceruloplasmin, was increased significantly on all the days following NDMA administration (Table 2). Lysyl oxidase is a copper-dependent enzyme involved in collagen cross-linking. The increased liver copper levels observed in the present study could be attributed to the increased synthesis and deposition of collagen during hepatic fibrosis.

Increased iron content was reported in the liver tissue of patients with alcoholic cirrhosis^44^. Hepatic iron overload is frequently observed in ALD patients and it is an important and independent factor for disease progression, survival, and the development of primary liver cancer^45^. It was reported that the iron overload during hepatic fibrosis is involved on collagen biosynthesis, cell proliferation, and expression of MMP-2 in rat hepatic stellate cells^17^. In addition, it was noticed that chronic hepatic iron overload is associated with hepatocellular injury, fibrosis, and ultimate cirrhosis^46,47^. The exact molecular mechanism of the deposition of hepatic iron during cirrhosis and ALD is not clear and could be attributed to decreased hepcidin. Acute exposure to alcohol drastically suppresses hepcidin, which could explain the ultimate accumulation of hepatic iron^48^. There was no alteration in serum iron levels in patients with alcoholic liver cirrhosis at various stages of the disease according to Child-Pugh classification^49^. In the present study also, we have not observed any changes in serum iron level during pathogenesis of hepatic fibrosis.

Manganese is very important for a number of biological functions especially in collagen and glycoprotein metabolism^20,50^. Increased serum manganese levels have been reported in patients with liver cirrhosis^27,51^. Elevated manganese levels in the hepatic tissue was observed in patients with chronic ALD^41^. In the current investigation also, we have noticed increased manganese levels in both serum and hepatic tissue following NDMA administration. The increased manganese levels in hepatic fibrosis may be due to increased intestinal absorption. It was reported that, in patients with hepatic cirrhosis, the manganese absorption is twice than in normal adults^52^.

Reports are not available regarding molybdenum levels during pathogenesis of hepatic fibrosis or alcoholic cirrhosis. In the present study, a significant increase was observed in serum molybdenum levels on 14th and 21st days following NDMA administration. In contrast, molybdenum levels were remarkably reduced on 14th and 21st days in the liver during NDMA treatment. The dramatic reduction of molybdenum concentration in the liver indicates that some factor is responsible for the mobilization of hepatic molybdenum into circulation. Interactions of molybdenum with other elements, especially iron and copper, could be playing a role.

Zinc has important role in connective tissue metabolism and wound healing^53,54^. Furthermore, zinc is an essential cofactor for over 200 metalloenzymes and involved in membrane protection against oxidants, synthesis of RNA and DNA, and glycoprotein metabolism^55^. Decreased levels of serum zinc have been well documented in hepatic fibrosis and alcoholic cirrhosis^11,49,51^. In addition, marked reduction in hepatic zinc content was reported in patients with various stages of liver diseases including cirrhosis^41,56^. The metabolic abnormalities resulting from zinc deficiency and the useful effects of zinc therapy has been evaluated in patients with chronic liver diseases^57^. We have noticed increased activity of tissue inhibitors of metalloproteinases (TIMP-1 and TIMP-2) in NDMA-induced hepatic fibrosis in rats^58^. Binding of TIMPs to zinc ion could contribute reduction of serum zinc levels. In the present investigation, both serum and hepatic zinc content were markedly decreased during the pathogenesis of NDMA-induced hepatic fibrosis. One of the major reasons for zinc depletion in hepatic fibrosis and alcoholic liver cirrhosis is decreased serum albumin levels. Albumin is the major zinc carrier and about 80% plasma zinc is bound to albumin^25^. In the current study we have observed a significant decrease of serum albumin levels on all the days following NDMA treatment. In alcohol abuse, alcohol-induced modulation of zinc transporters contribute decreased zinc levels in lungs, liver, gut, and brain^59^. The other factors of decreased serum and hepatic zinc content in liver diseases are inadequate dietary intake, impaired absorption, and enhanced clearance through urinary excretion^26^.

In conclusion, the results of the present study suggest that the modulation of trace elements during pathogenesis of hepatic fibrosis is correlated with metabolic imbalance, biochemical abnormalities, decreased serum albumin, and ascites following NDMA-induced liver injury. The alteration of trace elements during hepatic fibrosis could play a significant role in progression of the disease.
